# Comparative genomic analysis of *Staphylococcus aureus* isolates associated with either bovine intramammary infections or human infections demonstrates the importance of restriction-modification systems in host adaptation

**DOI:** 10.1099/mgen.0.000779

**Published:** 2022-02-18

**Authors:** Soyoun Park, Dongyun Jung, Bridget O’Brien, Janina Ruffini, Forest Dussault, Alexis Dube-Duquette, Élodie Demontier, Jean-François Lucier, François Malouin, Simon Dufour, Jennifer Ronholm

**Affiliations:** ^1^​ Faculty of Agricultural and Environmental Sciences, Macdonald Campus, McGill University, Québec, Canada; ^2^​ Mastitis Network, Saint-Hyacinthe, Québec, Canada; ^3^​ Regroupement FRQNT Op+Lait, Saint-Hyacinthe, Québec, Canada; ^4^​ Heath Canada, Ottawa, Ontario, Canada; ^5^​ Département de biologie, Faculté des sciences, Université de Sherbrooke, Sherbrooke, Canada; ^6^​ Faculté de Médecine Vétérinaire, Université de Montréal, Saint-Hyacinthe, Québec, Canada

**Keywords:** *Staphylococcus aureus*, bovine mastitis, comparative genomics, restriction-modification system

## Abstract

*
Staphylococcus aureus* is a major etiological agent of clinical and subclinical bovine mastitis. The versatile and adaptative evolutionary strategies of this bacterium have challenged mastitis control and prevention globally, and the high incidence of *S. aureus* mastitis increases concerns about antimicrobial resistance (AMR) and zoonosis. This study aims to describe the evolutionary relationship between bovine intramammary infection (IMI)-associated *S. aureus* and human pathogenic *S. aureus* and further elucidate the specific genetic composition that leads to the emergence of successful bovine IMI-associated *S. aureus* lineages. We performed a phylogenomic analysis of 187 *S *. *aureus* isolates that originated from either dairy cattle or humans. Our results revealed that bovine IMI-associated *S. aureus* isolates showed distinct clades compared to human-originated *S. aureus* isolates. From a pan-genome analysis, 2070 core genes were identified. Host-specific genes and clonal complex (CC)-specific genes were also identified in bovine *S. aureus* isolates, mostly located in mobile genetic elements (MGEs). Additionally, the genome sequences of three apparent human-adapted isolates (two from CC97 and one from CC8), isolated from bovine mastitis samples, may provide an snapshot of the genomic characteristics in early host spillover events. Virulence and AMR genes were not conserved among bovine IMI-associated *S. aureus* isolates. Restriction-modification (R-M) genes in bovine IMI-associated *S. aureus* demonstrated that the Type I R-M system was lineage-specific and Type II R-M system was sequence type (ST)-specific. The distribution of exclusive, virulence, and AMR genes were closely correlated with the presence of R-M systems in *S. aureus *, suggesting that R-M systems may contribute to shaping clonal diversification by providing a genetic barrier to the horizontal gene transfer (HGT). Our findings indicate that the CC or ST lineage-specific R-M systems may limit genetic exchange between bovine-adapted *S. aureus* isolates from different lineages.

## Data Summary

1. Short read data for bovine IMI-associated *S. aureus* isolates are available at NCBI-SRA under BioProject numbers PRJNA609123 and PRJNA622791.
2. Highly assembled human-originated *S. aureus* genomes used in this study are available at NCBI under the accession numbers listed in Additional file S1 (available in the online version of this article).
3. Metadata including source, collection year, geographical area, associated disease, and ST/CC, is summarized in Additional files S1 and S2.

**Impact Statement**

*
S. aureus* bovine mastitis is a costly disease in dairy cattle. Despite its clinical importance and overall burden in the dairy industry, studies of this bacterium isolated from cows have suffered due to a limited number of whole-genome sequences being available and a further limited subset of strains that can be genetically manipulated. Fully understanding *S. aureus* R-M systems may help explain HGT in this species and understand the dissemination of MGEs containing important virulence or AMR genes. Moreover, an understanding of *S. aureus* R-M systems may aid in designing strategies to bypass genetic barriers to make hyper recipients in a lineage of interest for genetic engineering applications. This approach may help facilitate studies on *S. aureus *, providing an improved understanding of its pathogenicity in a specific host.

## Introduction

*Staphylococcus aureus* is an opportunistic pathogen that can infect humans as well as economically important livestock such as cows, sheep, and goats. Among livestock, cows are a common reservoir of *S. aureus,* and dairy cattle frequently experience clinical and subclinical mastitis due to *S. aureus* intramammary infections (IMIs) [1]. *S. aureus* produces biofilms, survives in non-phagocytic and phagocytic host cells, and dynamically switches its phenotypes between wild-type and small colony variants [2–4]. Each of these characteristics results in the persistence of *S. aureus* colonization in intramammary environments. This persistence within the mammary glands typically leads to treatment failure and recurrent bovine mastitis. Bovine mastitis control programmes have been developed with the intention of infection prevention; however, knowledge gaps in clonal diversity, host immune response, and other elements that affect *S. aureus* IMIs hinder the development of effective prevention strategies [5]. Moreover, robust genetic defence mechanisms owing to restriction-modification (R-M) systems in *S. aureus* impede genetic manipulations, limiting researchers from understanding bovine IMI-associated *S. aureus* physiology, metabolism, and pathogenesis [6]. Thus, *S. aureus* remains a primary etiological agent of bovine IMIs, causing significant challenges for researchers, dairy farmers, and veterinarians.

On a short evolutionary time-scale, *S. aureus* lineages are host-specific; although, on a longer time-scale, lineages often undergo zoonosis and zooanthroponosis. The word *lineage* is used in this study to refer to a group of isolates that have a commonality either through being from the same sequence type (ST) or clonal complex (CC). Bovine-adapted *S. aureus* lineages include CC97, CC133, and CC151 and human-adapted lineages include CC1, CC5, CC8, CC30, and CC45 [7]. *S. aureus* host spillover, followed by adaptation to a new host, is generally accompanied by loss of virulence and immune evasion genes from the previous host and acquisition of a new set of genes specific for survival in the new host [8]. Whole-genome sequencing and a subsequent comparative genomic analysis can be used to understand the movement of virulence and other host adaptation genes between *S. aureus* isolates from different host species. Recent comparative genomic studies have shown that bovine-adapted strains rapidly lose genes involved in human infections, which likely increases their fitness in bovine hosts [8, 9]. For instance, bovine-specific mobile genetic elements (MGEs) found in bovine adapted *S. aureus* lineages include the temperate phages φSaBov and φPV83, as well as pathogenicity islands SaPlbov1, SaPlbov2, and SaPlbov3 [7, 9, 10]. These MGEs carry bovine-specific virulence factors such as LukMF’ and vWbp [11, 12].

Cows are a source of antimicrobial-resistant (AMR) *S. aureus *, which may be transferred to persist in humans [8]. Specifically, livestock-associated methicillin-resistant *S. aureus* (LA-MRSA) is believed, at least partially, to be responsible for community-associated MRSA due to the use of antimicrobials in veterinary medicine and modern agriculture [13]. Interestingly, the prevalence of AMR *S. aureus* among dairy cows differs significantly based on geography. In North America, for example, a low prevalence (less than 10%) of *blaZ* positive *S. aureus* has been reported, while China, Finland, Sweden, Iran, and Brazil have noted that 50–94 % of bovine *S. aureus* isolates are penicillin-resistant [14–19]. Most studies investigate only common STs or the prevalence of AMR genes, making it challenging to elucidate the links between genetic lineage, virulence, AMR, and adaptations to the bovine niche. Very few studies have attempted to correlate lineage and AMR to gain insight into this relationship. Among *S. aureus* CC97 isolated from cows, which includes several STs (ST97, ST115, and ST352), only a few STs are reported to be positive for *blaZ* or *mecA* [20–22]. Some human-adapted lineages, such as ST5 (CC5), ST8 (CC8), and their variants have been isolated from cows and were positive for *blaZ* and *mecA* [20–22]. These studies support the idea that certain *S. aureus* lineages may be more prone to obtaining specific AMR genes, suggesting that lineage-specific factors might be involved in horizontal gene transfer (HGT). The existence of *S. aureus* lineage-specific R-M systems is a possible explanation for the unequal distribution of virulence/AMR genes in different lineages from the bovine niche.

Acquisition of foreign DNA is important in terms of bacterial evolution, fitness, adaptation, and clonal diversification. Many virulence, host-specific, and AMR genes are carried by *S. aureus* MGEs, and the movement of these MGEs contributes to strain differentiation. Indeed, 15 % of any *S. aureus* genome consists of MGEs which play a prominent role in host adaptation and pathogenicity [23, 24]. However, acquiring foreign DNA is not always advantageous due to the possibility of obtaining harmful, lethal, or superfluous genes. To control the retention of foreign DNA some bacteria including *S. aureus* have developed R-M systems. R-M systems are grouped into four types based on subunit architecture, the requirement for ATP/GTP, the level of sequence specificity, and DNA cleavage mechanism [25]. Lineage-specific R-M systems with the combination of Type I, II, III, and IV R-M genes control the spread of clinically important genes between *S. aureus* CCs, with Type I and II systems being the most common [23, 26, 27]. Type I R-M systems are multi-subunit complexes that consist of two M subunits, two R subunits, and one S subunit, encoded by *hsdM*, *hsdR*, and *hsdS*, respectively [25]. The S subunit is responsible for recognizing a specific DNA sequence, the R subunit cleaves DNA, and the M subunit catalyses the methylation reaction [25]. Type I R-M systems are located in νSaα and νSaβ as a part of the core genome in *S. aureus *. The Type I R-M system is the primary R-M system in *S. aureus *, the alleles present are lineage-specific, and it constitutes a significant barrier to the movement of MGEs and intentional genetic manipulation [28]. Type II R-M systems consist of a restriction endonuclease (*res*), that recognizes a specific DNA sequence and then introduces double-strand DNA breaks, and a methyltransferase that recognizes the same DNA sequence and methylates it (*mod*) [29]. Methylation modifies and thus protects target DNA from cleavage by hiding it from the restriction endonuclease [29]. Type II R-M systems are widely used in recombinant DNA technology and because of this application more than 3500 have been discovered and characterized [30]. Type III R-M systems are also composed of two genes, *mod* and *res* that also function in DNA modification or restriction, respectively [25]. Type IV R-M systems are less well characterized, but are composed of one or two genes that encode proteins that cleave only modified sequences [25]. Understanding *S. aureus* lineage-specific genetic barriers can outline the HGT network and potential evolutionary directions, which would aid in understanding *S. aureus* and ultimately preventing *S. aureus* infections and dissemination of AMR genes. Furthermore, this knowledge enables us to improve our ability to manipulate non-transformable *S. aureus* for the purposes of future *S. aureus* studies.

In this study, we used a comparative genomics approach to investigate several aspects of bovine IMI-associated *S. aureus *. We attempted to further understand the evolutionary relationships between *S. aureus* isolated from humans and cows by identifying unequally distributed genes among the two hosts, as well as correlations between the presence of mastitis-associated virulence factors, AMR genes, and R-M system genes in bovine IMI-associated *S. aureus *.

## Methods

### Sequence genomes, assembly, and gene annotation

We previously reported whole-genome sequencing on bovine IMI-associated *S. aureus* isolates obtained from the Mastitis Pathogen Culture Collection (Additional file S1) [31–33]. Each of these isolates was obtained from the cows in different health status (Additional file S2). The raw DNA sequences of bovine isolates (*n*=63) were assembled following the same pipeline as previously described using the software pipeline ProkaryoteAssembly (v. 0.1.6) (https://pypi.org/project/ProkaryoteAssembly/) [31, 34]. The quality of the genome assemblies (draft genomes) was evaluated using Qualimap (v. 2.2.2) [35]. Complete genomes of human *S. aureus* isolates (*n*=122) and two reference genomes, RF122 and Newbould 305 isolated from a bovine milk samples, were obtained from the National Centre for Biotechnology Information (NCBI) database (Additional file S1). All *S. aureus* genomes (*n*=187) were then run through the annotation pipeline via Prokka (v. 1.14.5) with the genus/species option [36]. All draft and complete genomes were verified as *S. aureus* by confirming the presence of *crtOPQMN* operon and the binding site of *unc* universal primers [37, 38].

### Pan-genome and phylogenomic tree

A pan-genome of 187 *S *. *aureus* genomes was created using Roary (v. 3.13.0) with no paralog splitting and R plots options [39]. The pan-genome analysis was restricted to identifying the presence and absence of orthologs only; thus, paralogs copies were not taken into account during the analysis. The gene_presence_absence.csv file generated by Roary was used for pan-genome analysis. Core genes (core and soft core genes) and accessory genes (shell and cloud) were also identified using Roary.

The core gene alignment with 187 *S *. *aureus* genomes established by Roary was used to obtain phylogenetic estimates using IQ-TREE ModelFinder with 1000 replicate bootstraps [40]. The best model was found to be GTR+F+R2, which was then used to construct a phylogenomic tree and later displayed by iTOL (https://itol.embl.de/) [41]. We analysed seven housekeeping genes (*arcC, aroE, glpF, gmk, pta, tpi,* and *yqiL*) from each *S. aureus* genome to determine ST using mlst (https://github.com/tseemann/mlst) against the PubMLST database [42].

### Exclusive gene analysis

The genes from bovine and human *S. aureus* genomes were compared using Venny (v. 2.1.0) using the list of genes from the gene_presence_absence.csv file generated by Roary [43]. The genes only present in either bovine or human *S. aureus* were classified as exclusive genes. Within bovine *S. aureus *, lineage-specific genes were examined by comparing the genes present in each CC. The relative location of each exclusive gene or gene cluster was determined by aligning the draft genomes to the complete/reference genome of *S. aureus* RF122 (ET3-1) isolated from bovine using IslandViewer 4 [9, 44]. All exclusive or lineage-specific genes annotated as hypothetical or unknown functions from Roary were also searched on NCBI BLASTP using their amino acid sequences to identify potential functions [45].

### Identification of virulence/antimicrobial resistance/R-M genes

Virulence factors were analysed using VFanalyzer (http://www.mgc.ac.cn/VFs/main.htm) and confirmed with gene_presence_absence.csv file produced by Roary [46]. Antimicrobial resistance genes were analysed using ABRicate (https://github.com/tseemann/abricate) through MEGARes database and Roary [39, 47]. R-M genes were searched on Restriction-ModificationFinder 1.1 (https://cge.cbs.dtu.dk/services/Restriction-ModificationFinder/) and then analysed with REBASE searching through amino acid sequences and then grouped with >95 % amino acid sequence homology [48]. We classified *agr* types based on the conserved regions of amino acid sequences in AgrD (AIP precursor) [49]. The presence of the partially assembled or non-assembled genes was confirmed by Sanger sequencing followed by PCR with the target gene-specific primers (Additional file S3).

### Mobile genetic elements (MGEs) identification

Plasmids were analysed using NCBI blast initially with circular contigs from assembled sequences of bovine IMI-associated *S. aureus* isolates and then using ABRicate through the PlasmidFinder database [50]. The verified plasmids were run through a local blast with the parameter of 97 % identity and coverage against 65 bovine IMI-associated *S. aureus* genomes. Prophage and genomic islands sequences were identified using PHASTER and IslandViewer 4, respectively [44, 51]. All bovine IMI-associated *S. aureus* draft genomes were aligned against the bovine-adapted *S. aureus* RF122 (ET3-1) genome in IslandViewer 4 [9, 44].

## Results

### Phylogenomic tree and *S. aureus* pan-genome

**Fig. 1.**
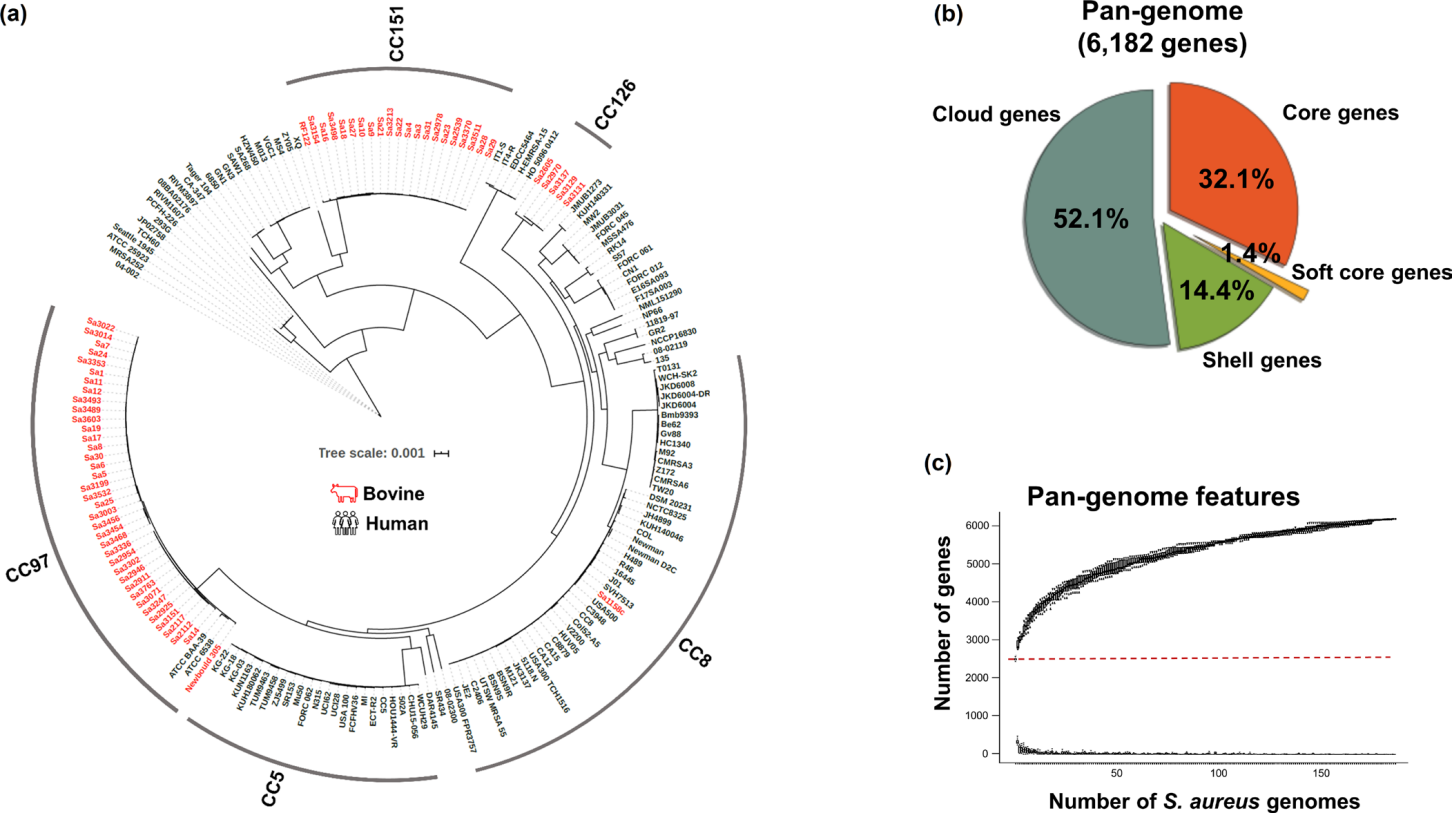
Phylogenomic tree and pan-genome of 187 *S *. *aureus* from human and bovine origins. (**a**) All bovine IMI-associated *S. aureus* isolates except Sa1158c (CC8) were clustered into three main clades: CC151, CC126, and CC97. (**b**) The pan-genome of 187 *S *. *aureus* isolates were subdivided into four groups: core (genes present in 99 % ≤isolates ≤100 %), soft core (95 % ≤isolates <99 %), shell (15 % ≤isolates <95 %), and cloud genes (0 % ≤isolates <15 %). (**c**) The red dotted line divides the graph into two features: total pan-genome in size (top) and the number of new genomes added to the total pan-genome (bottom) as new *S. aureus* genomes are added. The graph indicates an open state, and new genes are likely to be discovered continually as new genomes are added to the analysis.

Phylogenomic analysis was conducted using a core gene alignment and revealed that bovine IMI-associated *S. aureus* lineages included CC151, CC8, CC126, and CC97, and the majority of human isolates belonged to CC8 and CC5 (Fig. 1a). Bovine IMI-associated *S. aureus* isolates were clustered into three clades and distinct from human isolates. Three suspicious isolates that show evidence of having recently jumped betwen host species were CC8 (Sa1158c) and CC97 (ATCC BAA-39 and ATCC 6538).

All 489 086 coding sequences (CDS) from 187 *S *. *aureus* genomes were grouped into 6182 gene clusters. Grouping of the CDS revealed that the core genome to be 2070 genes (33.5 %) shared by more than 95 % of isolates and the accessory genome to be composed of 4112 genes (66.5 %) (Fig. 1b). The pan-genome increased in size upon the addition of new genomes suggesting an open pan-genome (Fig. 1c).

### Unequally distributed genes between clonal complexes

**Fig. 2.**
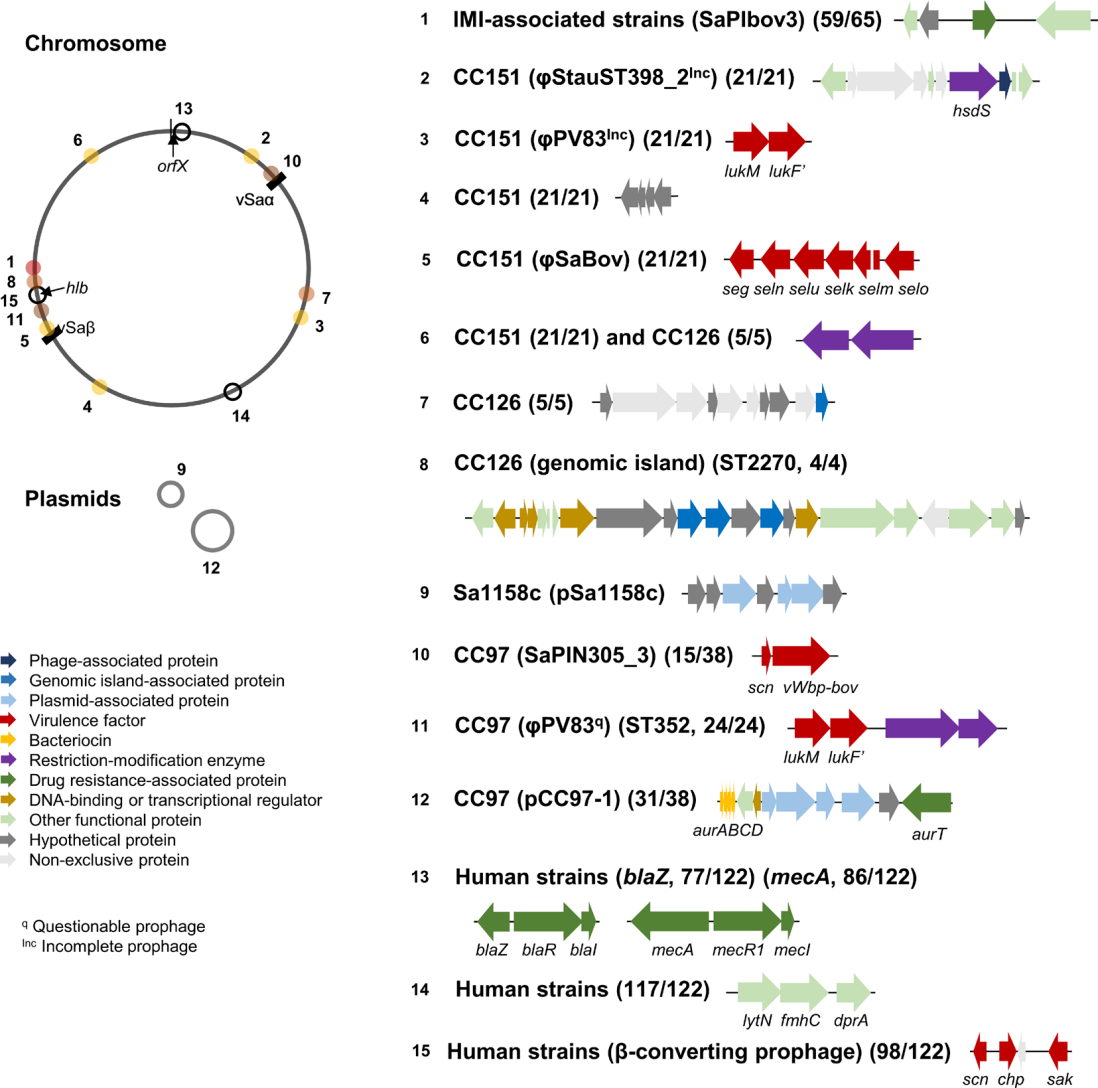
Distribution of lineage-specific genes in *S. aureus *. This figure illustrates the genes present mainly in bovine IMI-associated *S. aureus* isolates. The unequally distributed genes were shown with their associated lineages, MGEs, and frequency. A reference genome, *S. aureus* RF122, was used to identify the relative location of these genes in the genome as indicated by the corresponding numbers (1-15).

Unique and exclusive genes were examined, with a concentration on identifying genes that were unique to bovine IMI-associated *S. aureus* isolates. From the total pan-genome, 326 and 2653 elements were exclusive in either bovine IMI-associated or human *S. aureus* isolates, respectively. Most exclusive genes were either isolate-specific or CC lineage-specific. The most variable loci were found near *orfX* – the location of SCC*mec* integration in human isolates – and *hlb* (β-hemolysin) due to β-converting prophages and adjacent MGEs (Fig. 2).

Among bovine IMI-associated *S. aureus *, >90 % (59/65) of isolates contained a pathogenicity island known as SaPIbov3, which encodes four common proteins: a class I SAM-dependent methyltransferase, a hypothetical protein, a multidrug transporter, and a CAAX protease immunity-related protein. These unique genes found in bovine IMI-associated isolates were absent in five CC126 isolates and in one CC8 isolate (Sa1158c). We also found *lukMF’*, bovine-specific virulence genes, in CC151 and ST352 (CC97). All isolates in CC151 encoded common lineage-specific genes at five different loci: an incomplete prophage close to φStauST398, *lukMF’* genes carried by φPV83, four genes in a non-MGE region, an enterotoxin gene cluster located in φSaBov, and two genes encoding Type II R-M subunits in a putative genomic island. The same Type II R-M genes in CC151 were found in CC126. CC126 also had five unique genes, the functions of which were unclear. All ST2270 (CC126) isolates (*n*=4) contained 20 unique genes within the lineage located in a putative genomic island near *hlb*. Sa1158c (ST8/CC8) possessed its own plasmid that encoded seven open reading frames (ORFs) with unknown functions. Among the CC97 isolates, 15 isolates mainly ST2187 (*n*=13) carried bovine variants *vWbp* and *scn* located in a pathogenicity island. The *vWbp* and *scn* genes, associated with bovine immune evasion and commonly found in *S. aureus* isolated from cows, were rarely found in human isolates – only two human isolates (PCFH-226 and S58) encoded them. Additionally, 81.5 % (31/38) of CC97 isolates carried the pCC97-1 plasmid, which encodes the *aurABCD*, *aurI*, *aurR,* and *aurT* genes for aureocin synthesis and transportation.

Additionally, exclusive genes were also found in human-originated *S. aureus* isolates. Three genes: *lytN*, *fmhC*, and *dprA* associated with cell division, cell wall protection, and DNA processing, respectively, were present in most human isolates (95.9 %, 117/122) as a part of the core genome near *sucCD*, and these genes were found in only one bovine isolate Sa1158c (ST8), which was phylogenetically related to the human-adapted lineage. The majority of human isolates encoded an immune evasion cluster (80.3 %, 98/122) at the *hlb* locus: *scn*, *chp*, and *sak*. The enterotoxin gene cluster found in CC151 was also present among human isolates (*n*=45).

### Genetic elements suggesting recent host spillover

Three isolates which might be part of recent host spillover events (ATCC 6538, ATCC BAA-39, and Sa1158c) were identified in the phylogenomic tree (Fig. 1a). To identify potential genetic elements that might help *S. aureus* to enhance fitness or adaptation in a new host species, we further examined the presence of host-specific genes in these three isolates. One caveat to this analysis is that human and bovine isolates were derived from different geographic regions, which may confound the analysis.

ATCC 6538 and ATCC BAA-39 were isolated from humans and belong to ST464 (CC97). The four genes commonly found in bovine isolates (*n*=59) were absent in these two isolates. However, two bovine specific prophages similar to φPT1028 (incomplete) and φJS01 (intact) were found in these two isolates. A partial φPT1028 prophage that is frequently found in bovine IMI-associated *S. aureus* isolates (64.6 %, 42/65) was found; however, the version of φPT1028 found in the ATCC6538 and ATCC BAA-39 human isolates contained an additional pathogenicity island encoding enterotoxin genes (*entK* and *entQ*) that is not present in the version of φPT1028 that is associated with bovine isolates. These two enterotoxin genes; however, were also present in 34.4 % (42/122) of human-associated *S. aureus* isolates. Another prophage common to bovine isolates, φJS01, carried *scn* and *sak* and was inserted into *hlb* locus. The isolates ATCC 6538 and ATCC BAA-39 shared one *hsdS* with the bovine CC97 isolates, and showed defective Type I R-M genes, indicating a possible diverged evolutionary path via HGT (Additional file S5, Fig. S1).

Sa1158c (ST8), which was isolated from a bovine sample, encoded neither bovine-specific virulence genes nor the human immune evasion cluster carried by β-converting prophages. Sa1158c contained pSa1158c, a plasmid that encoded seven ORFs, but no known host adaptation genes. The pSa1158c plasmid was only found in the Sa1158c isolate, and did not have high sequence homology to any other plasmid. Type I R-M system genes were present, as with other ST8 isolates, although the presence of one of the *hsdM* genes was not confirmed due to incomplete genome assembly.

### Distribution of R-M systems and clonal diversification

**Fig. 3.**
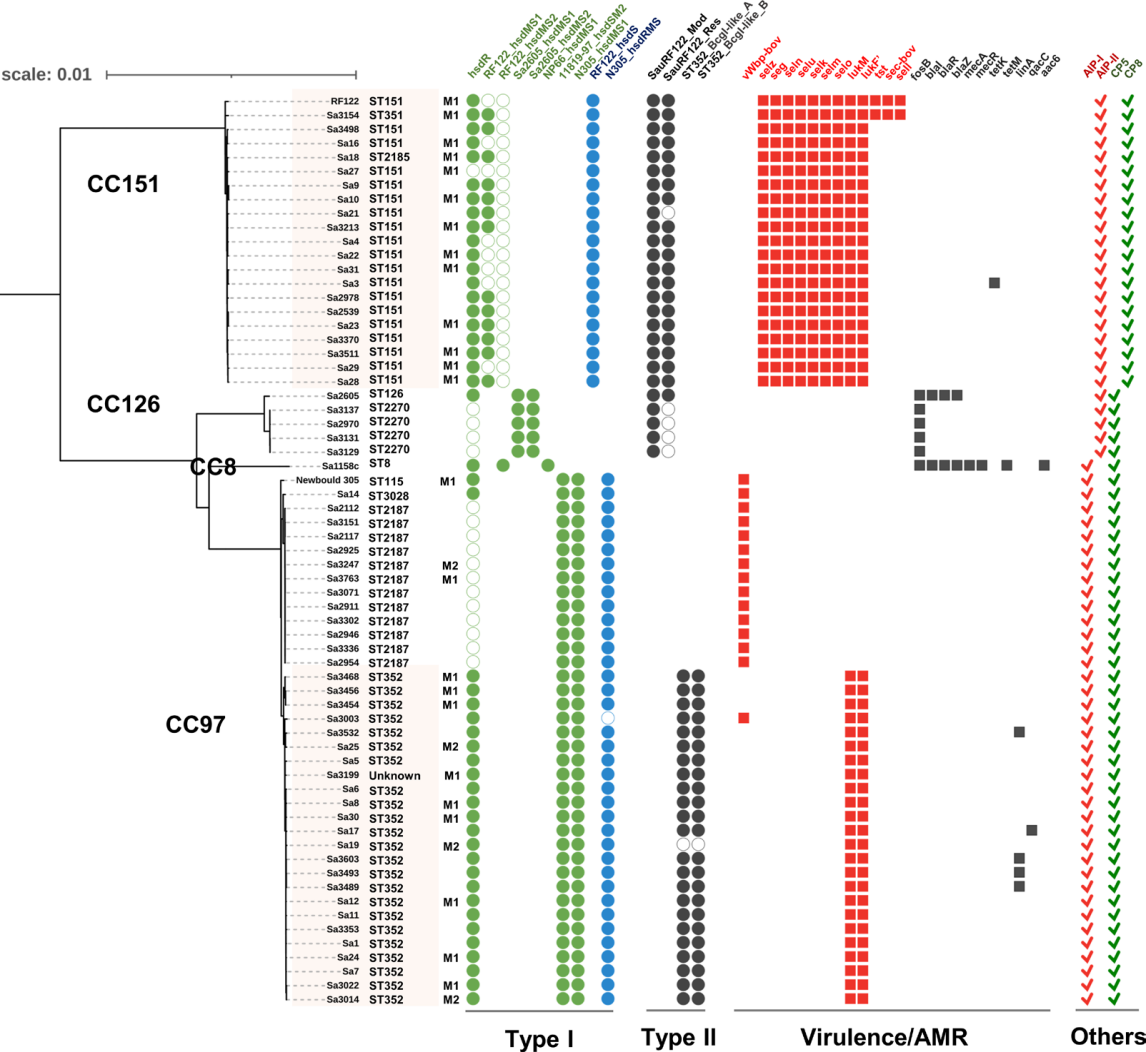
Distribution of restriction-modification genes and virulence/AMR genes in bovine IMI-associated *S. aureus *. The phylogenomic tree of 65 bovine IMI-associated *S. aureus* and relevant genetic content show lineage-specific R-M genes. Virulence and AMR genes are either ST-specific or isolate-specific. In the Type I R-M system, the green circular boxes indicate the Type I R-M system genes (*hsdR* and *hsdMS*) that are located in νSaα and νSaβ and are part of the core genome. The blue circular boxes show additional Type I R-M hsdRMS genes which are not a part of the *S. aureus* core genome. The *hsdR* and *hsdM* genes are highly conserved within the *S. aureus* species; however, several *hsdS* alleles exist. While each of the *hsdMS* genes shown in green are interchangeable, part of the same R-M system, and should, in combination with the *hsdR*, form a functional complex, the Type I R-M system genes shown in blue would not be expected to be interchangeable, and would instead form a separate, and independent Type I R-M system. The black circular boxes represent Type II R-M genes that encode a pair of enzymes: a methyltransferase and a restriction endonuclease. The red and the black square boxes represent virulence and AMR genes, respectively. The open circular boxes in all shapes and colours indicate the presence of a pseudogene. The red and green checkmarks indicate signal molecule (AIP-I and AIP-II) and capsular polysaccharide (CP5 and CP8). The highlighted isolates were associated with clinical mastitis. M1 is used to indicate that the milk sample was collected on the day clinical mastitis was diagnosed, and M2 indicates the sample was collected 14 days after a diagnosis of clinical mastitis was made. An extended report of each of the R-M genes identified in all isolates included in this study can be found in Fig. S1.

Using RESBASE database, four R-M systems (Type I, II, III and IV) were found among the 187 *S *. *aureus* genomes. Both human and bovine IMI-associated *S. aureus* isolates mainly possessed Type I and/or II R-M systems and only few human isolates carried Type III/IV R-M genes (Additional file S5, Fig. S1). As a part of the core genome, the Type I R-M system is a primary barrier to free HGT, and the combination of two *hsdS* gene copies was found to be lineage-specific. In bovine IMI-associated *S. aureus* isolates, Type II R-M system seemed to be more ST-specific (Fig. 3). In human *S. aureus* isolates, R-M systems in MGEs were either isolate-specific or ST-specific (Additional file S5, Fig. S1).

Bovine isolates had unique *hsdS* genes in two genomic islands (νSaα and vSaβ) and HsdS with >95 % amino acid sequence homology was only found in two ST80 human isolates (11819–97 and GR2). However, the combination of two Type 1 R-M system *hsdS* genes was unique in bovine-adapted STs. The two major bovine CCs (CC151 and CC97) carried unique *hsdS* alleles*,* which provide specificity to the Type I R-M enzymes. In CC151, the *hsdS* gene in vSaβ was truncated and additional *hsdS* genes were found in a prophage, likely to aid the primary Type I R-M system owing to high amino acid homology of this additional *hsdS* with the primary Type I *hsdS* found in other STs . All bovine CC97 isolates carried two sets of Type I R-M systems. Unlike the original Type I R-M genes, an additional *hsdRMS* locus was located near *orfX* (Additional file S5, Fig. S1). In ST2187 (CC97), due to the mutation in *hsdR* the primary Type I R-M was inactivated, yet the presence of additional *hsdRMS* suggested it replaced the original Type I R-M system in this ST.

All bovine isolates, except Sa1158c, possessed Type II R-M genes encoding two subunits (Mod and Res) in putative genomic islands. Although CC151 and CC126 shared the same Type II R-M genes, defective genes were found in SauRF122 Res subunit in all ST2270 isolates. Interestingly, Type II R-M genes in ST352 (CC97) were found right beside bovine-specific virulence genes *lukMF’*, suggesting that dissemination of these virulence genes is likely to be limited within ST352 and closely related STs.

### Bovine IMI-associated *S. aureus* virulence factors and AMR genes in MGEs

To better understand the correlation between the distribution of virulence/AMR genes and *S. aureus* R-M systems, we examined virulence/AMR genes (Fig. 3). Due to the lack of information regarding the chronological acquisition order of virulence/AMR and R-M genes, we only investigated their distribution. Their causal relationships were not taken into account in this analysis.

We identified a total of 103 virulence genes from 65 bovine IMI-associated *S. aureus* isolates, which included adhesions (*n*=17), enzymes (*n*=15), immune evasion elements (*n*=23), secretion system (*n*=12), and toxins (*n*=36) (Additional file S4). Most virulence genes were conserved in all bovine isolates as a part of the core genome. It is noteworthy that the ST2187 isolate (CC97), in which the *hsdR* was inactivated, carried a bovine variant gene encoding for a von Willebrand factor-binding protein (vWbp). Several toxin genes (*n*=12) were located in MGEs and showed uneven distribution between STs. All CC151 isolates contained *selz* near *orfX* and an enterotoxin gene cluster (*seg*, *seln*, *selu*, *selk*, *selm*, and *selo*) in an intact prophage φSaBov closest to φIpla88. On the contrary, isolates from CC97 which had two *hsdRMS* sets were found to carry no enterotoxin gene cluster. We also found Sa3154 contained a pathogenicity island encoding three virulence factors (*tst*, *sec*, and *sell*) that was integrated adjacent to νSaα. Among CC97 isolates, only ST352 carried *lukMF’* in a putative prophage closest to φPV83, which contained Type II R-M genes for BcgI-like alpha/beta subunits serving as a self-restricted MGE. Interestingly, no CC lineage-specific virulence gene was found in CC126 although their Type I and II R-M genes were inactivated.

A total of 25 AMR genes were found using MEGARes database and Roary from 65 *S *. *aureus* isolated from bovine sources (Additional file S4). Of the 25 AMR genes, majority encoded efflux pumps (*n*=10) and regulators (*n*=7). A total of 14 genes were found in all bovine IMI-associated *S. aureus* isolates examined in this study: *aac*(3), *aph*(3), *arlR*, *arlS*, *pbuE*, *lmrS*, *mepA*, *mepB*, *slyA*, *mgrA*, *norA*, *norB*, *rlmH* and *tet*(38). The remaining AMR genes (*n*=11) in bovine isolates were located in MGEs, with the exception of *fosB*. Unlike the virulence genes identified, no AMR genes exclusive to bovine isolates were found. All CC126 isolates (*n*=5) had *fosB*, and among them, only Sa2605 (CC126) carried *blaI*, *blaR*, and *blaZ*. In addition to the 14 core AMR genes, Sa1158c (CC8) contained eight more AMR genes: *fosB* in non-MGE, *mecR* and *mecA* at *orfX*, *blaI* and *blaZ* at the downstream of *SCCmec*, *aacA* in a genomic island, and *tetM* in another genomic island. Only Sa3 (CC151) among bovine IMI-associated isolates carried *tetK* in pSX10B1. Four isolates in ST352 (CC97) were found with *linA* carried by plasmids (Fig. 3). The plasmids with *linA* were rarely present in human isolates (2.5 %, 3/122). Instead of carrying bovine-specific virulence genes, CC126 and CC8 isolated from bovine niche carried AMR genes which were highly restrained within the STs and not found in other STs. Unlike virulence genes, correlations between *S. aureus* R-M system and AMR genes were not found.

### Gene content involved in bovine mastitis in non-MGEs

We additionally investigated *agr* genes and capsular biosynthesis genes that are associated with *S. aureus* quorum-sensing and pathogenesis (Fig. 3). We investigated the autoinducing peptide (AIP) type in STs. Only AIP-I and II were found in bovine isolates, while all four known AIP types were found in human isolates; although only a minority of isolates (13.1%, 16/122) encoded either the AIP-III or AIP-IV precursor (Additional file S5, Fig. S2). All CC151 isolates encoded the AIP-I precursor, and others had *agrD* for the AIP-II precursor. The capsular biosynthesis gene cluster *capABCDEFGHIJKLMNOP* in CC151 was different from the other bovine isolates, specifically in four genes (*capHIJK*). CC151 encoded *cap8HIJK* while others carried *cap5HIJK*, as known that two capsular types are produced by bovine IMI-associated *S. aureus* isolates.

## Discussion

In this study, we highlighted the pan-genome of 187 *S *. *aureus* isolates obtained from bovine and humans and compared their gene contents. Human *S. aureus* isolates (*n*=122) contributed significantly to the pan-genome, which was mainly from the accessory genes. The origin of the isolates, collection period and area, and quality of the assembled genomes likely influence the pan-genome size [52, 53]. The accessory genomes of bovine and human isolates were composed of 1375 and 3771 genes, respectively. *S. aureus* genomes originated from humans used in this study were from various geographical areas over a broad range of collection years, while bovine *S. aureus* isolates were mainly collected for 2 years in Canada (Additional file S1). Moreover, the completeness of *S. aureus* genomes from humans was much better than bovine IMI-associated *S. aureus* genomes.

We examined host-specific genes of *S. aureus* isolated from both humans and cows. *S. aureus* host-specifity is closely correlated to genetic lineage – CC97, CC126, CC133, and CC151 are bovine adapted lineages, while human-adapted lineages include CC1, CC5, CC8, CC30, and CC45 [7]. The pathogenicity island known as SaPIbov3 carries four bovine-specific genes and has been reported to be exclusively found in bovine isolates [54]. In this study, SaPIbov3 was not found in bovine isolates from either the CC126 or CC8 lineages. Similarly, human-specific genes such as *lytN, fmhC, dprA*, *scn*, *chp*, and *sak* were encoded within the majority of human STs, yet not all human isolates. The distribution of these host-specific genes suggests that there is no absolute or universal host-specific element, although they can increase an opportunity for the successful adaptation in a new host niche. Alternatively, losing human-specific MGEs and acquiring a single mutation may also confer a fitness advantage and alter host tropism [55, 56].

From the phylogenomic tree, we found three suspicious host jumping isolates: ATCC 6538 and ATCC BAA-39 in CC97 and Sa1158c in CC8. Zoonotic and zooanthroponotic transfers of *S. aureus* between humans and cows in both CC97 and CC8 have been reported in other studies [8, 21, 57]. It is still speculative whether these three *S. aureus* isolates truly result from the host-switching between humans and cows. However, the genetic elements in these isolates could provide an insight into the potential host jumping route or mechanism of other isolates in the same CC. The two human isolates (CC97), ATCC 6538 and ATCC BAA-39, carried two enterotoxin genes in a pathogenicity island and the immune evasion cluster located in β-converting prophage φJS01, which are highly associated with virulence and host-adaptation [58, 59]. Thus, these two CC97 isolates seem to be successfully adapted to the new host by acquiring this human immune evasion cluster. We previously observed that plasmid transformation from RN4220 (CC8) to CC97 more efficient than those from CC8 to CC151 under laboratory conditions [60, 61]. This indicates CC97 is more prone to be accept genes from human-originated *S. aureus* than CC151, increasing its chance to survive in human niche. On the contrary, no host-specific gene was found in bovine-isolated Sa1158c (CC8). However, the loss of a beta-converting prophage has shown to be associated with human-to-bovine jump of *S. aureus *. The possibility of spillover and transmission of CC8 from human to bovine is still valid in this specific isolate and more zooanthroponotic transfer is possible via the same manner under the right selection pressure while repeated exposure of CC8 to bovine occurs.

Most genes exclusive to bovine-associated *S. aureus* were located within MGEs. These MGEs explain that clonal diversification of *S. aureus* may occur via HGT during the adaptation to the bovine niche. The differences between CC97 and CC151, two major bovine lineages, in MGEs were enterotoxin gene cluster in CC151 and *vWbp* for bovine-specific coagulase in CC97. Although CC151 encoded more toxin genes than CC97, we found *tst* located in SaPIbov in RF122 and Sa3154, probably due to ST bias to ST151 that was previously reported not to carry SaPIbov [62]. We showed that all bovine-associated *S. aureus* had Type I R-M genes with a unique combination of *hsdS* genes in different lineages, and many bovine isolates also carried Type II R-M genes. This lineage- or ST-specific genetic barrier suggests that R-M systems in *S. aureus* are, at least in part, responsible for shaping clonal diversification. The original and additional Type I R-M systems are unlikely to form an interchangeable functional complex due to the low amino acid identity between the various subunits. This additional HsdRMS may play a critical role in ST2187 (CC97) due to a defective *hsdR* in the original complex and an overall enhanced genetic barrier in CC97. ST2270 (CC126) may be a restriction-defective ST with no known functional R-M system due to the inactivated HsdR (Type I R-M) and Res subunit (Type II R-M). However, restriction endonuclease deficient *S. aureus* strains are not necessarily hypersusceptible to gene transfer. We observed that ST2270 isolates did not carry more MGEs than other STs. It was previously demonstrated that the inactivation of Type I R-M system was insufficient to construct *S. aureus* mutants capable of efficiently accepting foreign DNA [63]. *S. aureus* may naturally develop another barrier for gene transfer because lacking R-M system is more vulnerable to bacteriophage suggesting its detrimental effect over beneficial effect [64].

Of note, *S. aureus* R-M systems are not an absolute barrier for gene transfers. Under certain environmental pressure, increased dissemination of MGEs can occur within a lineage or across different lineages. Exposure to antibiotic pressure is one stimulator of genetic dissemination since antibiotic-induced SOS response promotes HGT of pathogenicity islands [65]. This HGT network raises concerns regarding the dissemination of AMR genes from human to bovine hosts via host transmission we described in this study. Sa1158c (ST8) carrying *blaZ* and *mecA* is a potential donor of AMR genes to *S. aureus* in the bovine niche. AMR genes (*mecA* and *blaZ*) have been identified in ST97 (CC97) and ST126 (CC126) in Brazil, suggesting that right selective pressure may overcome or bypass the genetic barrier to disseminate AMR genes in bovine-adapted lineages [66]. *S. aureus* ST97 is a MRSA lineage extensively found in pigs and dairy cattle in Italy [67]. Although ST97 shares the same Type I R-M genes with other STs of CC97 [68], it does not carry Type II R-M genes that are present in ST352, making HGT from ST97 to ST352 more challenging than other STs of CC97. However, the HGT network of AMR genes in bovine-adapted lineage CC97 is already open under the right selective pressure.

Interestingly, we also observed antagonistic characteristics of CC151 and CC97 against each other. CC151 and CC97 encoded *agrD* for different AIP precursors involved in *S. aureus* quorum-sensing activity. AIP-I and II produced by *S. aureus* are known to exhibit cross-inhibition [49]. Indeed, we previously confirmed that CC151 (*agr* type II) inhibited the quorum-sensing of CC97 (*agr* type I) in co-culture conditions [60]. The pCC97-1 plasmid, which is most homologous to pRJ80 plasmid with 99.78 % identity, was found in CC97 isolates and encoded genes for aureocin 4181 products: aureocin peptides, known as heat-stable bacteriocins, bacteriocin immunity protein, bacteriocin regulatory protein, and bacteriocin exporter protein [69]. Aureocin 4181 is known to exhibit strong antimicrobial activity against isolates of *Micrococcus luteus *, *Streptococcus agalactiase*, *S. aureus *, and other staphylococci [69]. This bacteriocin may modify the microbial composition of the udder skin and teat canal thus disturbing the native microbiome. We also observed that CC97 carrying pCC97-1 plasmid inhibited the growth of CC151 *in vitro* [60]. The antagonistic relationship within bovine-adapted *S. aureus* lineages (CC97 and CC151) suggests that they have evolved independently and are unlikely to dominate the same host at the same time point.

The main limitation of this study was the lack of information on the chronological order of the acquisition of MGEs and R-M genes in *S. aureus *, leading to a failure to elucidate the causal relationships between them. Also, sequence recognition sites of each R-M enzyme commonly found in bovine IMI-associated *S. aureus* are unknown, so the presence of cognition sequences in MGEs could not be determined. An additional limitation of this study was from the incomplete draft genomes of bovine IMI-associated *S. aureus *. It is often recommended to use a draft genome with a ‘near finished’ status (less than 1 % missing fraction) in pan-genome computations [52]. Apart from common criteria such as GC%, N50, and a number of contigs, the genome size and exclusion of draft genomes smaller than 2.65 Mb, corresponding to <97 % of *S. aureus* RF122 genome size, was performed in this study. Some important genes in a few isolates were not assembled, yet the genes were still present. To confirm the presence of target genes, such as *hsdMS*, PCR amplification and Sanger sequencing needed to be performed. The presence of plasmids was also confirmed by plasmid DNA extraction and mapping to verify the size of the predicted plasmids. Lastly, 63 bovine IMI-associated *S. aureus* isolates used in this study give a bias to Canada while human isolates were from various continents. This strong geographical bias may result in misleading the data interpretation such as CC lineage-specific exclusive genes.

## Conclusion

The genetic differences among bovine IMI-associated *S. aureus* lineages reveal that *S. aureus* in the bovine niche has evolved in multiple directions. Our results suggest that bovine-specific and exclusive genes, which are mainly located in MGEs, play an important role in clonal diversification and host adaptation. Moreover, R-M systems in *S. aureus* shape *S. aureus* clonal diversification and pathogenicity by discriminating MGEs. We highlight that *S. aureus* ST identification in dairy herds is important to assess the risk of transmission and intervention strategies due to the various potential impacts of certain STs on dairy cows. We also bring attention to the possible MRSA transmission from humans to cows, suggesting the continued importance of farm biosecurity.

## Supplementary Data

Supplementary material 1
