# Supplementary material for: Comparative genomic analysis of Staphylococcus aureus isolates associated with either bovine intramammary infections or human infections demonstrates the importance of restriction-modification systems in host adaptation
Source: Microb Genom. 2022 Feb 18;8(2):000779. doi: 10.1099/mgen.0.000779 (PMC8942034; doi:10.1099/mgen.0.000779)
Supplement: Supplementary material 1 [file mgen-8-0779-s001.pdf]

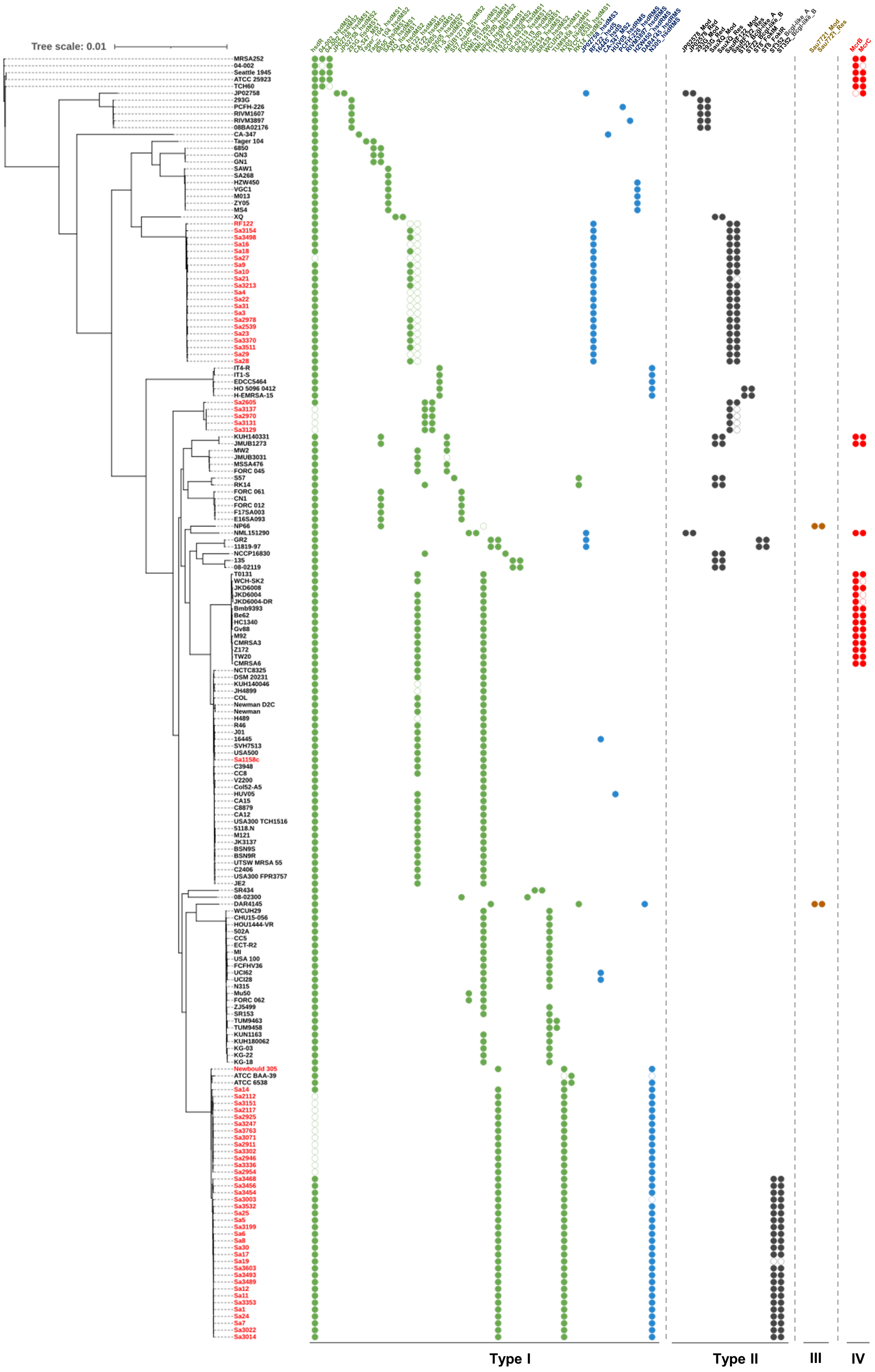
Tree scale: 0.01
Type I
Type II
III
IV

Fig. S1. Distribution of restriction-modification genes in 187 *S. aureus*. The phylogenomic tree of 187 *S. aureus* and aligned R-M genes show the lineage-specific distribution of the R-M genes. Both human and bovine IMI-associated S. aureus isolates mainly possessed TypeI/II R-M systems and only few human isolates carried TypeIII/IV R-M genes. In the Type I R-M system, the green circular boxes indicate the Type I R-M system genes (hsdR and hsdMS) located in νSaα and νSaβ as part of the core genome. The blue circular boxes show additional Type I R-M hsdRMS genes which are not a part of the *S. aureus* core genome. While each of the *hsdMS* genes shown in green are interchangeable, part of the same R-M system, and should, in combination with the *hsdR*, form a functional complex, the Type I R-M system genes shown in blue would not be expected to be interchangeable, and would instead form a separate, and independent Type I R-M system. The isolates in red in the phylogenomic tree were originated from bovine. The colored circles represent the presence of the R-M genes, and the open circles indicate the inactivated R-M genes.

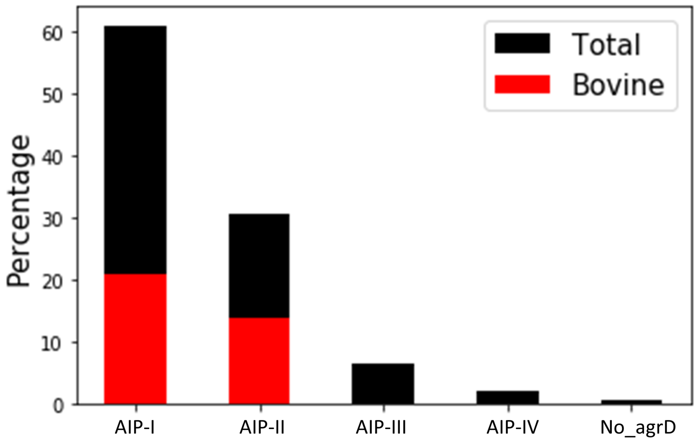

Fig. S2. Distribution of AIPs in 187 *S. aureus*. AIP-I precursor was the most prevalent in *S. aureus* in this study, followed by AIP-II. AIP-I and II were found in bovine isolates (n = 65), while all four known AIP types were found in human isolates (n = 122). Among human isolates, only small fraction of the population encoded AIP-III (n = 13) and AIP-IV (n = 4).

Additional File 1

No.	Strain name	Accession no. NCBI	Genome structure	Country of origin	Host	Disease/clinical information	ST	CC	year	Seq.
1	Sa1	JAANBU000000000	Draft genome	Canada	Bovine	Intramammary infection	ST352	CC97	2007	I
2	Sa3	JAANBR000000000	Draft genome	Canada	Bovine	Intramammary infection	ST151	CC151	2007	I
3	Sa4	JAANCG000000000	Draft genome	Canada	Bovine	Intramammary infection	ST151	CC151	2007	I
4	Sa5	JAANCB000000000	Draft genome	Canada	Bovine	Intramammary infection	Unknown	Unknown	2007	I
5	Sa6	JAANCH000000000	Draft genome	Canada	Bovine	Intramammary infection	ST352	CC97	2007	I
6	Sa7	JAANBN000000000	Draft genome	Canada	Bovine	Intramammary infection	ST352	CC97	2007	I
7	Sa8	JAANCD000000000	Draft genome	Canada	Bovine	Intramammary infection	ST352	CC97	2008	I
8	Sa9	JAANBZ000000000	Draft genome	Canada	Bovine	Intramammary infection	ST151	CC151	2007	I
9	Sa10	JAANCE000000000	Draft genome	Canada	Bovine	Intramammary infection	ST151	CC151	2007	I
10	Sa11	JAANCA000000000	Draft genome	Canada	Bovine	Intramammary infection	ST352	CC97	2007	I
11	Sa12	JAANCC000000000	Draft genome	Canada	Bovine	Intramammary infection	ST352	CC97	2008	I
12	Sa14	JAANCF000000000	Draft genome	Canada	Bovine	Intramammary infection	ST3028	CC97	2007	I
13	Sa16	JAANBM000000000	Draft genome	Canada	Bovine	Intramammary infection	ST151	CC151	2007	I
14	Sa17	JAANBL000000000	Draft genome	Canada	Bovine	Intramammary infection	ST352	CC97	2007	I
15	Sa18	JAANBK000000000	Draft genome	Canada	Bovine	Intramammary infection	ST2185	CC151	2008	I
16	Sa19	JAANBW000000000	Draft genome	Canada	Bovine	Intramammary infection	ST352	CC97	2008	I
17	Sa21	JAANBX000000000	Draft genome	Canada	Bovine	Intramammary infection	ST151	CC151	2007	I
18	Sa22	JAANBY000000000	Draft genome	Canada	Bovine	Intramammary infection	ST151	CC151	2007	I
19	Sa23	JAANBP000000000	Draft genome	Canada	Bovine	Intramammary infection	ST151	CC151	2007	I
20	Sa24	JAANBS000000000	Draft genome	Canada	Bovine	Intramammary infection	ST352	CC97	2007	I
21	Sa25	JAANBO000000000	Draft genome	Canada	Bovine	Intramammary infection	ST352	CC97	2007	I
22	Sa27	JAANBI000000000	Draft genome	Canada	Bovine	Intramammary infection	ST151	CC151	2019	I
23	Sa28	JAANBH000000000	Draft genome	Canada	Bovine	Intramammary infection	ST151	CC151	2019	I
24	Sa29	JAANBG000000000	Draft genome	Canada	Bovine	Intramammary infection	ST151	CC151	2019	I
25	Sa30	JAANBF000000000	Draft genome	Canada	Bovine	Intramammary infection	ST352	CC97	2019	I
26	Sa31	JAANBE000000000	Draft genome	Canada	Bovine	Intramammary infection	ST151	CC151	2007	I
27	Sa1158c	SRR11471981	Draft genome	Canada	Bovine	Intramammary infection	ST8	CC8	2008	I
28	Sa2112	SRR11471976	Draft genome	Canada	Bovine	Intramammary infection	ST2187	CC97	2007	I
29	Sa2117	SRR11471989	Draft genome	Canada	Bovine	Intramammary infection	ST2187	CC97	2007	I
30	Sa2539	SRR11471965	Draft genome	Canada	Bovine	Intramammary infection	ST151	CC151	2007	I
31	Sa2605	SRR11471994	Draft genome	Canada	Bovine	Intramammary infection	ST126	CC126	2008	I
32	Sa2911	SRR11471986	Draft genome	Canada	Bovine	Intramammary infection	ST2187	CC97	2008	I
33	Sa2925	SRR11471988	Draft genome	Canada	Bovine	Intramammary infection	ST2187	CC97	2007	I
34	Sa2946	SRR11471975	Draft genome	Canada	Bovine	Intramammary infection	ST2187	CC97	2007	I
35	Sa2954	SRR11471977	Draft genome	Canada	Bovine	Intramammary infection	ST2187	CC97	2007	I
36	Sa2970	SRR11471973	Draft genome	Canada	Bovine	Intramammary infection	ST2270	CC126	2007	I
37	Sa2978	SRR11471964	Draft genome	Canada	Bovine	Intramammary infection	ST151	CC151	2008	I
38	Sa3003	SRR11471960	Draft genome	Canada	Bovine	Intramammary infection	ST352	CC97	2007	I
39	Sa3014	SRR11471958	Draft genome	Canada	Bovine	Intramammary infection	ST352	CC97	2008	I
40	Sa3022	SRR11471957	Draft genome	Canada	Bovine	Intramammary infection	ST352	CC97	2007	I
41	Sa3071	SRR11471985	Draft genome	Canada	Bovine	Intramammary infection	ST2187	CC97	2008	I
42	Sa3129	SRR11471990	Draft genome	Canada	Bovine	Intramammary infection	ST2270	CC126	2008	I
43	Sa3131	SRR11471991	Draft genome	Canada	Bovine	Intramammary infection	ST2270	CC126	2008	I
44	Sa3137	SRR11471992	Draft genome	Canada	Bovine	Intramammary infection	ST2270	CC126	2008	I
45	Sa3151	SRR11471971	Draft genome	Canada	Bovine	Intramammary infection	ST2187	CC97	2008	I
46	Sa3154	SRR11471968	Draft genome	Canada	Bovine	Intramammary infection	ST351	CC151	2007	I
47	Sa3199	SRR11471956	Draft genome	Canada	Bovine	Intramammary infection	ST352	CC97	2008	I
48	Sa3213	SRR11471970	Draft genome	Canada	Bovine	Intramammary infection	ST151	CC151	2007	I
49	Sa3247	SRR11471974	Draft genome	Canada	Bovine	Intramammary infection	ST2187	CC97	2008	I
50	Sa3302	SRR11471972	Draft genome	Canada	Bovine	Intramammary infection	ST2187	CC97	2008	I
51	Sa3336	SRR11471993	Draft genome	Canada	Bovine	Intramammary infection	ST2187	CC97	2008	I
52	Sa3353	SRR11471959	Draft genome	Canada	Bovine	Intramammary infection	ST352	CC97	2007	I
53	Sa3370	SRR11471963	Draft genome	Canada	Bovine	Intramammary infection	ST151	CC151	2008	I
54	Sa3454	SRR11471984	Draft genome	Canada	Bovine	Intramammary infection	ST352	CC97	2008	I
55	Sa3456	SRR11471983	Draft genome	Canada	Bovine	Intramammary infection	ST352	CC97	2007	I
56	Sa3468	SRR11471982	Draft genome	Canada	Bovine	Intramammary infection	ST352	CC97	2008	I
57	Sa3489	SRR11471978	Draft genome	Canada	Bovine	Intramammary infection	ST352	CC97	2008	I
58	Sa3493	SRR11471962	Draft genome	Canada	Bovine	Intramammary infection	ST352	CC97	2008	I
59	Sa3498	SRR11471966	Draft genome	Canada	Bovine	Intramammary infection	ST151	CC151	2007	I
60	Sa3511	SRR11471969	Draft genome	Canada	Bovine	Intramammary infection	ST151	CC151	2007	I
61	Sa3532	SRR11471980	Draft genome	Canada	Bovine	Intramammary infection	ST352	CC97	2007	I
62	Sa3603	SRR11471979	Draft genome	Canada	Bovine	Intramammary infection	ST352	CC97	2008	I
63	Sa3763	SRR11471987	Draft genome	Canada	Bovine	Intramammary infection	ST2187	CC97	2007	I
64	RF122	NC_007622	Draft genome	Ireland	Bovine	Intramammary infection	ST151	CC151	1993	
65	Newbould 305	NZ_AKYW01000001 - NZ_AKYW01000028	Highly assembled	Canada	Bovine	Clinical mastitis	ST115	CC97	1958	I
66	04-002	NZ_CP038021	Highly assembled	USA	Human	Abdominal wound	ST30	CC30	2004	PB
67	08-02119	NZ_CP015645	Highly assembled	Germany	Human	Wound infection	ST582	CC15	2008	PB, I
68	08-02300	NZ_CP015646	Highly assembled	Germany	Human	Wound infection	ST7		2008	PB, I
69	08BA02176	NC_018608	Highly assembled	Canada	Human	LA-MRSA origin, soft tissue infection of skin	ST398		2008	R
70	11819-97	NC_017351, NC_017350	Highly assembled	Denmark	Human	CA-MRSA, skin abscess	ST80		1997	I, R
71	135	NZ_CP022720, NZ_CP022721	Highly assembled	Germany	Human	Bacteremia	ST15	CC15	1994	I, PB
72	16445	NZ_CP043302, NZ_CP043303	Highly assembled	USA	Human		ST8	CC8	2019	O
73	293G	NZ_CP019591	Highly assembled	Canada	Human		ST398			I, PB
74	502A	NZ_CP007454, NZ_CP007455	Highly assembled	USA	Human	Colonization	ST5	CC5	1963	PB
75	5118.N	NZ_CP016855, NZ_CP016854, NZ_CP025482	Highly assembled	USA	Human	Nose from military trainee	ST8	CC8	2010	I
76	6850	NC_022222	Highly assembled		Human	Bacteremia	ST50			R, SG
77	ATCC_25923	NZ_CP009361, NZ_CP009362	Highly assembled	USA	Human		ST243	CC30	1945	PB
78	ATCC_6538	NZ_CP020020, NZ_CP020021	Highly assembled	Germany	Human	Pleural fluid	ST464	CC97	1884	PB, I
79	ATCC_BAA-39	NZ_CP033505	Highly assembled	Kazakhstan	Human		ST464	CC97	2017	PB
80	BSN9R	NZ_CP042348, NZ_CP042349	Highly assembled	USA	Human	Infective endocarditis	ST8	CC8	2018	O, I
81	BSN9S	NZ_CP042346, NZ_CP042347	Highly assembled	USA	Human	Infective endocarditis	ST8	CC8	2018	O, I
82	Be62	NZ_CP012013, NZ_CP012014	Highly assembled	Brazil	Human	Blood stream infection	ST239	CC8	1996	R
83	Bmb9393	NC_021670.1, NC_021657.1	Highly assembled	Brazil	Human	Blood stream infection	ST239	CC8	1993	R
84	C2406	CP019590	Highly assembled	Canada	Human	USA300, Necrotizing pneumonia	ST8	CC8		PB
85	C3948	CP020957	Highly assembled	Canada	Human	MSSA	ST8	CC8		PB
86	C8879	NZ_CP020956	Highly assembled	Canada	Human	MRSA	ST8	CC8		PB
87	CA-347	NC_021554, NC_021552	Highly assembled	USA	Human	USA600	ST45	CC45	2005	I, PB
88	CA12	NZ_CP007672, NZ_CP007673	Highly assembled	Colombia	Human	Bacteremia	ST8	CC8	2007	PB
89	CA15	NZ_CP007674, NZ_CP007675	Highly assembled	Colombia	Human	Bacteremia	ST8	CC8	2007	PB
90	CC5	NZ_CP021105	Highly assembled	Brazil	Human	Respiratory tract infection	ST5	CC5	2014	I
91	CC8	NZ_AP017377	Highly assembled	Russia	Human		ST8	CC8	2007	PB
92	CHU15-056	NZ_CP021171	Highly assembled	Brazil	Human	Bloodstream infection	ST5	CC5	2015	I
93	CMRSA3	CP029685, MH470063	Highly assembled	Canada	Human	MRSA	ST241	CC8		I, PB
94	CMRSA6	CP027788	Highly assembled	Canada	Human	MRSA	ST239	CC8		I, PB
95	CN1	NC_022226, NC_022227, NC_022228	Highly assembled	South Korea	Human	PVL-negative CA-MRSA	ST72	CC8		R
96	COL	NC_002951, NC_006629	Highly assembled	USA	Human	MRSA	ST250	CC8	1976	SG
97	Col52-A5	NZ_CP040560, NZ_CP040561	Highly assembled	Colombia	Human	Colonization	ST923	CC8	2017	PB
98	DAR4145	NZ_CP010526	Highly assembled	India	Human	MRSA	ST772	CC1	2009	PB
99	DSM_20231	NZ_CP011526, NZ_CP011527	Highly assembled		Human		ST8	CC8	1884	PB
100	E16SA093	NZ_CP031131	Highly assembled	South Korea	Human	Blood stream infection	ST72	CC8	2016	PB
101	ECT-R2	NC_017343, NC_017346, NC_017344	Highly assembled	Sweden	Human	MR-MSSA	ST5	CC5	2005	R
102	EDCC5464	NZ_CP022291	Highly assembled	Germany	Human	Implant associated bone infection	ST22	CC22	2015	PB
103	F17SA003	NZ_CP031130	Highly assembled	South Korea	Human	Blood stream infection	ST72	CC8	2017	PB
104	FCFHV36	NZ_CP011147	Highly assembled	Brazil	Human	Osteomyelitis	ST105	CC5	2010	I

Legend	
SG	Shotgun sequencing
PB	PacBio
I	Illumina
R	Roche (454)
SL	SOLiD
O	Oxford nanopore
IT	Ion Torrent
*	Unknown or not applicable
-	Unknown or not applicable
bold	Reference strains
y	Yes
n	No

105	FORC_012	NZ_CP010998	Highly assembled	South Korea	Human	Sputum	ST72	CC8	2009	I, PB
106	FORC_045	NZ_CP017115	Highly assembled	South Korea	Human		ST1	CC1	2014	PB
107	FORC_061	NZ_CP022607, NZ_CP022608	Highly assembled	South Korea	Human	Food poisoning	ST72	CC8	2017	PB
108	FORC_062	NZ_CP022582	Highly assembled	South Korea	Human	Food poisoning	ST5	CC5	2017	PB
109	GN1	NZ_AP018349	Highly assembled	Japan	Human	MSSA	ST50		2005	PB
110	GN3	NZ_AP017891	Highly assembled	Japan	Human	MSSA	ST50		2005	PB
111	GR2	NZ_CP010402, NZ_CP010403, NZ_CP010404	Highly assembled	Greece	Human		ST80		2006	R, I
112	Gv88	NZ_CP012018, NZ_CP012017	Highly assembled	Brazil	Human	Wound infection	ST239	CC8	1997	R
113	H-EMRSA-15	NZ_CP007659	Highly assembled	Belgium	Human	Abscess	ST22	CC22	2011	I
114	H489	NZ_CP020959	Highly assembled	Canada	Human	MSSA	ST8	CC8		PB
115	HC1340	NZ_CP012011	Highly assembled	Brazil	Human	Nasa colonization	ST239	CC8	2001	R
116	HOU1444-VR	NZ_CP012593, NZ_CP012594, NZ_CP012595, NZ_CP012596	Highly assembled	Brazil	Human	Bacteremia	ST5	CC5	2012	PB
117	HO_5096_0412	NC_017763	Highly assembled	UK	Human	Fatal neonatal infection	ST22	CC22	2005	I
118	HUV05	NZ_CP007676, NZ_CP007677, NZ_CP007678, NZ_CP007679	Highly assembled	Colombia	Human	Bacteremia	ST8	CC8	2006	PB
119	HZW450	NZ_CP020741	Highly assembled	China	Human	Impetigo	ST59		2016	PB
120	IT1-S	NZ_CP028468, NZ_CP028469	Highly assembled	Italy	Human	Endocarditis	ST22	CC22	2013	I
121	IT4-R	NZ_CP028470, NZ_CP028471	Highly assembled	Italy	Human	Endocarditis	ST22	CC22	2013	I
122	J01	NZ_CP040619, NZ_CP040620, NZ_CP040621	Highly assembled	USA	Human	Infective endocarditis	ST8	CC8	2011	I, PB
123	JE2	NZ_CP020619	Highly assembled	USA	Human	Soft tissue infections	ST8	CC8	2013	I, PB
124	JH4899	NZ_AP014921, NZ_AP014922	Highly assembled	Japan	Human	Invasive growth	ST8	CC8	2013	I
125	JK3137	CP020960	Highly assembled	Canada	Human	MRSA	ST8	CC8		PB
126	JKD6004-DR	NZ_CP040625	Highly assembled	USA	Human		ST239	CC8	2019	I
127	JKD6004	NZ_CP040622	Highly assembled	Australia	Human	Pacemaker abscess	ST239	CC8	2004	I, PB
128	JKD6008	CP002120	Highly assembled	New Zealand	Human	MRSA, VSSA	ST239	CC8	2003	R, SG, SL
129	JMUB1273	NZ_AP018922	Highly assembled	Japan	Human	Subcutaneous abscess	ST188	CC1	2016	I, O
130	JMUB3031	NZ_AP018923, NZ_AP018924	Highly assembled	Japan	Human	Subcutaneous abscess	ST1	CC1	2017	PB, I
131	JP02758	NZ_AP017922, NZ_AP017923	Highly assembled	Japan	Human	Super biofilm-elaborating	Unknown	Unknown	2005	PB
132	KG-03	NZ_AP019542	Highly assembled	Japan	Human	Persistent bacteremia	ST5	CC5	2015	PB, I
133	KG-18	NZ_AP019543, NZ_AP019544	Highly assembled	Japan	Human	VISA	ST5	CC5	2015	PB, I
134	KG-22	NZ_AP019545, NZ_AP019546	Highly assembled	Japan	Human	VISA	ST5	CC5	2015	PB, I
135	KUH140046	NZ_AP020313, NZ_AP020314	Highly assembled	Japan	Human	MRSA	ST8	CC8	2014	I, O
136	KUH140331	NZ_AP020316, NZ_AP020317	Highly assembled	Japan	Human	MRSA	ST188	CC1	2014	I, O
137	KUH180062	NZ_AP020320, NZ_AP020321	Highly assembled	Japan	Human		ST764	CC5	2018	I, O
138	KUN1163	NZ_AP020324, NZ_AP020325	Highly assembled	Japan	Human		ST764	CC5	2006	I, O
139	M013	NC_016928, NZ_CP039996	Highly assembled	Taiwan	Human	CA-MRSA	ST59		2002	R
140	M121	NZ_CP007670, NZ_CP007671	Highly assembled	Colombia	Human	Healthy volunteer	ST8	CC8	2004	I, PB
141	M92	CP015447	Highly assembled	Canada	Human	MRSA, Colonization	ST5354	CC8		PB
142	MI	NZ_AP017320, NZ_AP017321	Highly assembled		Human	VISA, Peritonitis	ST5	CC5	1997	I, PB
143	MRSA252	NC_002952	Highly assembled	UK	Human	MRSA, Fatal bacteremia	ST36	CC30	1997	
144	MS4	NZ_CP009828	Highly assembled	China	Human	Wound exudate of bone fracture	ST338		2012	I, PB
145	MSSA476	BX571857, BX571858	Highly assembled		Human	Osteomyelitis and bacteremia	ST1	CC1	1998	
146	MW2	BA000033, AP004832	Highly assembled	USA	Human	CA-MRSA, septicaemia and septic arthritis	ST1	CC1	1998	SG
147	Mu50	BA000017, AP003367	Highly assembled	Japan	Human	Surgical wound infection, VRSA	ST5	CC5	1997	SG
148	N315	BA000018, AP003139	Highly assembled	Japan	Human	Pharyngeal smear, *mecA* but methicillin susceptible	ST5	CC5	1982	
149	NCCP16830	NZ_CP043843, NZ_CP043844	Highly assembled	South Korea	Human	Diabetes, urine	ST513		2008	PB, IT
150	NCTC8325	NC_007795	Highly assembled		Human	Toxic-shock syndrome, staphylococcal scarlet fever	ST8	CC8		
151	NML151290	MEGZ00000001-MEGZ00000008	Highly assembled	Canada	Human	Endocarditis and septic arthritis	ST25			I
152	NP66	NZ_CP041037	Highly assembled	South Africa	Human	Pus aspirate	ST12		2018	PB
153	Newman	NC_009641	Highly assembled	UK	Human		ST254	CC8	1952	SG
154	Newman_D2C	NZ_CP023391	Highly assembled		Human	Osteomyelitis	ST254	CC8	1970	I
155	PCFH-226	NZ_CP035005, NZ_CP035006	Highly assembled	South Korea	Human	Healthy, hand	ST541		2017	PB
156	R46	NZ_CP039164, NZ_CP039165, NZ_CP039166	Highly assembled	Pakistan	Human		ST113	CC8	2017	I
157	RIVM1607	NZ_CP013619, NZ_CP013620	Highly assembled	Netherlands	Human		ST398		2008	R, I
158	RIVM3897	NZ_CP013621	Highly assembled	Netherlands	Human	LA-MRSA, nosocomial transmission	ST398		2008	R, I
159	RK14	NZ_CP011528, NZ_CP011529	Highly assembled	Germany	Human	Staphylococcal food poisoning	ST27	CC8	2008	I, PB
160	S57	NZ_CP030136	Highly assembled	Brazil	Human	Osteomyelitis	ST9	CC1	2010	PB
161	SA268	NZ_CP006630	Highly assembled	China	Human		ST59		2012	I
162	SAW1	NZ_CP045468, NZ_CP045469, NZ_CP045470, NZ_CP045471	Highly assembled	China	Human	Endocarditis	ST59		2018	O
163	SR153	NZ_CP048643, NZ_CP048644, NZ_CP048645, NZ_CP048646	Highly assembled	China	Human	Acute pancreatitis	ST5	CC5	2013	PB
164	SR434	NZ_CP019563, NZ_CP019564, NZ_CP019565, NZ_CP019566, NZ_CP019567	Highly assembled	China	Human	Skin abscess	ST88		2015	PB
165	SVH7513	NZ_CP029166, NZ_CP029167, NZ_CP029165	Highly assembled	Australia	Human	Cellulitis	ST612	CC8	2009	PB, I
166	Seattle_1945	NZ_CP021907, NZ_CP021908	Highly assembled	Germany	Human	PVL+	ST243	CC30	2013	I
167	T0131	NC_017347	Highly assembled	China	Human	MRSA	ST239	CC8	2006	R
168	TCH60	NC_017342, NC_017345,	Highly assembled		Human	MRSA	ST4618	CC30		R
169	TUM9458	NZ_AP019305	Highly assembled		Human		ST2389	CC5	2008	I, O
170	TUM9463	NZ_AP019306	Highly assembled		Human		ST2389	CC5	2009	I, O
171	TW20	NC_017331, NC_017352, NC_017332	Highly assembled	UK	Human	MRSA	ST239	CC8	2003	
172	Tager_104	NZ_CP012409	Highly assembled	USA	Human	Cutaneous abscess	ST49		1947	I, PB
173	UCI28	NZ_CP018768, NZ_CP018769	Highly assembled	USA	Human	No contact with swine	ST5	CC5	2009	PB, I
174	UCI62	NZ_CP018766, NZ_CP018767	Highly assembled	USA	Human	No contact with swine	ST5	CC5	2010	PB, I
175	USA300_FPR3757	CP000255, CP000256, CP000257, CP000258	Highly assembled	USA	Human	MRSA	ST8	CC8		SG
176	USA300_TCH1516	NC_010079, NC_010063, NC_012417	Highly assembled	USA	Human	CA-MRSA	ST8	CC8		R
177	USA500	NZ_CP007499, NZ_CP007500	Highly assembled	USA	Human		ST8	CC8		I, PB
178	USA_100	NZ_CP029474, NZ_CP029475	Highly assembled	USA	Human		ST5	CC5	2015	I, O
179	UTSW_MRSA_55	NZ_CP013231, NZ_CP013227, NZ_CP013228, NZ_CP013229, NZ_CP013230	Highly assembled	USA	Human	Osteomyelitis, MRSA	ST8	CC8	2013	PB
180	V2200	NZ_CP007657,	Highly assembled	Venezuela	Human	Osteomyelitis	ST923	CC8	2007	PB
181	VGC1	NZ_CP039448, NZ_CP039449, NZ_CP039450	Highly assembled	Taiwan	Human	Pneumonia and bacteremia	ST59		2013	I, O
182	WCH-SK2	NZ_CP031537	Highly assembled	Australia	Human	Pneumoniae, SSTI, Cystic fibrosis	ST239	CC8	2009	PB
183	WCUH29	NZ_CP039156	Highly assembled	Poland	Human	MRSA, Osteomyelitis	ST5	CC5	2004	PB
184	XQ	NZ_CP013137	Highly assembled	China	Human	Acute skin infections	ST121		2009	IT
185	Z172	NC_022604, NC_022610, NC_022605	Highly assembled	Taiwan	Human	VISA, Bacteremia	ST239	CC8	2010	I, PB
186	ZJ5499	NZ_CP011685	Highly assembled	China	Human	Pulmonary infection	ST5	CC5	2010	I
187	ZY05	NZ_CP045472, NZ_CP045473	Highly assembled	China	Human	Toxic shock syndrome	ST338		2016	PB

Additional File 2

Sample	Mastitis Asscoiation	SampleType	MastitisScore	SCC (x 1,000 cells/mL)
Sa6	No	L1	0	214
Sa4	No	L1	0	886
Sa14	No	L1	0	77
Sa10	Yes	M1	3	missing value
Sa8	Yes	M1	2	missing value
Sa12	Yes	M1	3	missing value
Sa5	No	V1	missing value	4133
Sa11	No	T1	0	445
Sa9	No	L2	0	11
Sa22	Yes	M1	1	missing value
Sa21	No	T1	0	231
Sa19	Yes	M2	missing value	missing value
Sa1	No	L1	0	1008
Sa24	Yes	M1	2	missing value
Sa3	No	L2	1	3534
Sa23	Yes	M1	2	missing value
Sa25	Yes	M2	missing value	4571
Sa7	No	T1	0	126
Sa16	Yes	M1	2	missing value
Sa17	No	T1	å	8483
Sa18	Yes	M1	1	missing value
Sa27	Yes	M1	2	missing value
Sa28	Yes	M1	1	missing value
Sa29	Yes	M1	2	missing value
Sa30	Yes	M1	1	missing value
Sa31	Yes	M1	3	missing value
Sa2112	No	L1	0	5162
Sa2605	No	T2	0	-2
Sa2946	No	T1	0	499
Sa2954	No	V1	0	2611
Sa2970	No	T1	0	3134
Sa2978	No	L1	0	1452
Sa3151	No	T1	0	1616
Sa3247	Yes	M2	0	2706
Sa3302	No	T2	0	6038
Sa3336	No	L3	0	928
Sa3489	No	L1	0	11
Sa3532	No	L3	missing value	149
Sa3603	No	L3	0	1713
Sa3763	Yes	M1	1	missing value
Sa3511	Yes	M1	1	missing value
Sa2539	No	L1	0	398
Sa3370	No	L1	0	3525
Sa3003	No	L1	0	2620
Sa3014	Yes	M2	missing value	47
Sa3199	Yes	M1	2	missing value
Sa3131	No	T1	0	2188
Sa2117	No	L1	0	404
Sa2911	No	L1	0	489
Sa3454	Yes	M1	1	missing value
Sa3468	Yes	M1	1	missing value
Sa3493	No	L1	0	11
Sa3353	No	L1	0	1273
Sa3022	Yes	M1	2	missing value
Sa3137	No	T1	0	1577
Sa3129	No	T1	0	198
Sa2925	No	L1	0	972
Sa3071	No	L1	0	9
Sa3456	Yes	M1	missing value	7159
Sa1158c	No	L2	0	440
Sa3213	Yes	M1	2	missing value
Sa3154	Yes	M1	2	missing value
Sa3498	No	L1	0	missing value

Legend	
M1	day of diagnostic
M2	+14 days after diagnostic
L1-L3	during lactation
T1 and T2	before drying-off
V1	after calving

Additional File 3

Primers	Nucleotides Sequence (5'-3')	Target gene
blaZ-F	CAAAGATGATATAGTTGCTTATTCTCC	*blaZ*
blaZ_R	TGCTTGACCACTTTTATCAGC	*blaZ*
hsdM_F	ATGTCTATTACTGAAAAACAACG	*hsdM*
hsdM_R	TTACTCATCTTTCAACACCC	*hsdM*
CC97_hsdSb_F	ATAAGAGTGATAAATTTAACCCTC	*hsdS*
CC97_hsdSb_R	GCTGCAATTCAATTAGTTTTTCATTTC	*hsdS*
USA500_hsdSa_F	AGATAGAGTAATTAGGAAAAATAAAAAC	*hsdS*
USA500_hsdSa_R	TTTTTTAATTGTTTATATTTTAAGTTCC	*hsdS*

Additional File 4

VFclass	Virulence factors	Related genes
Adherence	Autolysin	atl
	Cell wall associated fibronectin binding protein	ebh
	Clumping factor A	clfA
	Clumping factor B	clfB
	Elastin binding protein	ebp
	Fibrinogen binding protein	efb
	Fibronectin binding proteins	fnbA
		fnbB
	Intercellular adhesin	icaA
		icaB
		icaC
		icaD
		icaR
	Ser-Asp rich fibrinogen-binding proteins	sdrC
		sdrD
		sdrE
	Staphylococcal protein A	spa
Enzyme	Cysteine protease	sspB
		sspC
	Hyaluronate lyase	hysA
	Lipase	geh
		lip
	Serine V8 protease	sspA
	Serine protease	splA
		splB
		splC
		splD
		splE
		splF
	Staphylocoagulase	coa
	von Willebrand factor-binding protein	vWbp-bov
	Thermonuclease	nuc
Immune evasion	Capsule	capA
		cap8B
		cap8C
		cap8D
		cap8E
		cap8F
		cap8G
		cap5H
		cap5I
		cap5J
		cap5K
		cap8H
		cap8I
		cap8J
		cap8K
		cap8L
		cap8M
		capN
		cap8O
		cap8P

	AdsA	adsA
	SCIN	scn
	Sbi	sbi
Secretion system	Type VII secretion system	esxA
		esaA
		essA
		esaB
		essB
		essC
		esxC
		esxB
		essE
		esxD
		essD
		esaG
Toxin	Alpha hemolysin	hly/hla
	Beta hemolysin	hlb
	Delta hemolysin	hld
	Enterotoxin Z	selz
	Enterotoxin C bovine variant	sec-bov
	Enterotoxin G	seg
	Enterotoxin-like K	selk
	Enterotoxin-like L	sell
	Enterotoxin-like M	selm
	Enterotoxin-like N	seln
	Enterotoxin-like O	selo
	Enterotoxin-like U	selu
	Exfoliative toxin type A	eta
	Exotoxin	set11
		set16
		set17
		set18
		set19
		set20
		set21
		set22
		set23
		set24
		set25
		set26
		set30
		set34
		set36
	Gamma hemolysin	hlgA
		hlgB
		hlgC
	Leukotoxin M	lukM
	Leukotoxin F'	lukF'
	Leukotoxin D	lukD
	Leukotoxin E	lukE
	Toxic shock syndrome toxin	tst

Function	AMR factors	Related genes
Aminoglycosides resistance	AAC3	aac3
	AAC6-PRIME	aac6-prime

	APH3-PRIME	aph3-prime
MDR regulator	ARLR	arlR
	ARLS	arlS
Penicillin resistance	BLAI	blaI
	BLAR	blaR
	BLAZ	blaZ
Phenicol resistance	DHAP	dhaP
Fosfomycin resistance	FOSB	fosB
Biocide resistance	LMRS	lmrS
Lincosamide resistance	LNUA	linA
beta-lactam resistance	MECA	mecA
	MECR	mecR
Multi-drug resistance	MEPA	mepA
	MEPB	mepB
	MEPR	mepR
MDR regulator	MGRA	mgrA
Biocide resistance	NORA	norA
	NORB	norB
Biocide resistance	QACC	qacC
MLS resistance	RLMH	rlmH
Tetracycline resistance	TET38	tet38
	TETK	tetK
	TETM	tetM
